# *In Vitro* Cytotoxic Evaluation of a Novel Phosphinyl Derivative of Boldine

**DOI:** 10.3390/molecules16032253

**Published:** 2011-03-07

**Authors:** Franz A. Thomet, Pablo Piñol, Joan Villena, Patricio G. Reveco

**Affiliations:** 1 Department of Chemistry, Universidad Técnica Federico Santa María, Avenida España N° 1680, Valparaíso, Chile; E-Mail: franz.thomet@usm.cl (F.A.T.); 2 Centro Regional de Estudios en Alimentos Saludables (CREAS), Department of Biomedical Science, School of Medicine, Universidad de Valparaíso, Avenida Hontaneda N° 2664, Valparaíso, Chile; E-Mails: pignol@yahoo.com (P.P.); juan.villena@uv.cl (J.V.)

**Keywords:** synthesis, phosphinyl derivative, boldine, cytotoxic activity

## Abstract

2,9-Dimethoxymethylboldine (**2**), 2,9-dimethoxymethyl-3-bromoboldine (**3**) and 2,9-dimethoxymethyl-3-diphenylphosphinylboldine (**4**) have been synthesized in an effort to find compounds with potential pharmacological applications. The cytotoxic activities of the natural precursor **1** and these three derivatives have been measured as IC_50_ inhibitory growth. The diphenylphosphinyl derivative **4** showed a significant cytotoxic activity on two breast cancer cell lines, namely MCF-7 and MDA-MB-231, with IC_50_ values of 55.5 and 62.7 [µM], respectively. These results suggest that the kind of structural modifications introduced to synthesize compound **4** represent a promising way to enhance the cytotoxic activity of boldine derivatives.

## 1. Introduction

Boldine (**1**), the major alkaloidal constituent of the Chilean boldo tree (*Peumus boldus* Molina, Monimiaceae) has been extensively studied due to its anti-inflammatory, antipyretic and antioxidant activity (in both *in vitro* as well as *in vivo* models) [1], and derivatives of boldine with significant biological activity have already been reported in the literature [2]. A synthetic route that uses lithiation to introduce a different functional group on the aporphinic skeleton has been previously published by our research group [3].

The search for a potential biological application of the cytotoxicity of boldine has already yielded encouraging results. A methanolic leaf extract of *Peumus boldus* exhibiting cytotoxic activity on two types of human cancer cells [4], the demonstration of an intercalating agent behavior of boldine [5] and its antiproliferant activity against a glioma cell line [6] are examples of promising findings that motivate further research in this area.

In the search of coordination compounds involving the pharmacologically active metals Pt(II), Ru(II) and Au(I) and boldine, various groups were attached to the natural product to make possible the coordination. The present work focuses on the preparation of a new diphenylphosphinyl derivative **4** of boldine and the assessment of its *in vitro* inhibitory effect on the growth of two breast cancer lines (MDA-MB-231, MCF-7) and one dermal human fibroblast cell line (DHF). These inhibitory effects have been compared to those obtained for compounds **1**–**3**.

## 2. Results and Discussion

### 2.1. Chemistry

**Scheme 1:**
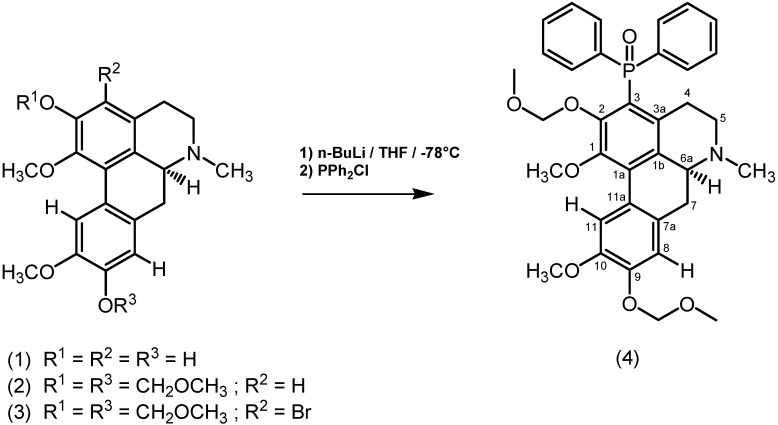
Boldine derivatives synthesized.

The new diphenylphosphinyl derivative **4** was prepared in good yield employing compound **3** as a precursor for the lithium-bromine exchange reaction (Scheme 1). 

The appearance of ten additional aromatic protons in the ^1^H-NMR spectrum associated to the diphenylphosphinyl group, and the strong upfield shift of the dioxymethylene protons on position C-2 from 5.24 and 5.33 ppm (*J* = 5.7 Hz) in **3** to 3.85 and 4.78 ppm (*J* = 4.9 Hz) in **4** indicated the substitution on the C-3 position. ^13^C-NMR (including dept-135, together with 1D-TOCSY and 2D HSQC/HMBC experiments) and ^31^P-NMR confirmed the structure proposed for **4**. Oxidation of the diphenylphosphine to the diphenylphosphinyl function was attributed to the chromatographic elution conditions (CHCl_3_/MeOH = 99/1), given that in the halo-dehydroxylation procedures PPh_3_ is employed [7]. This conclusion is supported by the MS parent ion at m/z 616.2481.

### 2.2. Cytotoxic Activity

To quantify the cytotoxic effect of boldine (**1**) and the synthetic derivatives **2**–**4**, the IC_50_ value of each compound was measured (the IC_50_ value is defined as the µM concentration of the compound that achieves a 50% reduction in cellular growth after 72 hours of drug exposure). Table 1 shows the IC_50_ values for compounds **1** through **4** for two types of breast cancer cell (MDA-MB-231 and MCF-7) and the DHF fibroblast cell line. The results clearly indicate that only the diphenylphosphinyl derivative **4** exhibits a significant cytotoxic activity, and that such activity emerges in the breast cancer cells.

**Table 1:** Cytotoxic activity (IC_50_) of boldine (**1**) and its synthetic derivatives **2–4**.

Compound	IC_50_ (µM)		
	MCF-7	MDA-MB-231	DHF
**1**	>100	>100	>100
**2**	>100	>100	>100
**3**	>100	>100	>100
**4**	55.5 ± 6.7	62.7 ± 4.8	>100

## 3. Experimental

### 3.1. General

NMR experiments were performed using an Avance 400 Digital Bruker NMR spectrometer, operating at 400.13 MHz for ^1^H, 100.61 MHz for ^13^C and 161.97 MHz for ^31^P. Chemical shifts (δ) are given in ppm and coupling constant (J) in Hz. ^1^H-NMR chemical shifts are relative to the proton resonance resulting from incomplete deuteration of CDCl_3_ (δ 7.26), those of ^13^C-NMR are relative to the carbon of the CDCl_3_ (δ 77.0) and those of ^31^P-NMR are relative to 85% H_3_PO_4_ external standard. High resolution mass spectrum was recorded by direct injection on a LTQ Orbitrap XL (Thermo Fischer Scientific) with electrospray ionization (ESI/MS) in positive mode. The data were acquired an analyzed using the software Xcalibur v. 2.0.7. Boldine (**1**) was extracted from *Peumus boldus* Molina using a established procedure. 2,9-Dimethoxymethylboldine (**2**) and 2,9-dimethoxymethyl-3-bromo-boldine (**3**) were prepared following a previously reported procedure [2,3]. Chlorodiphenylphosphine was freshly distilled prior to use. All reagents were obtained from Aldrich Co. and were used without further purification. Solvents were purchased from either J.T. Baker or Tedia Company Inc.

### 3.2. Chemistry: Synthesis of 2,9-Dimethoxymethyl-3-diphenylphosphinylboldine *(**4**)*

To a solution of 2,9-dimethoxymethyl-3-bromoboldine (**3**, 135 mg, 0.27 mmol) in dry THF (9 mL), cooled at −78 °C, n-BuLi (0.50 mL, 1.6 M, 0.80 mmol) was added under an inert atmosphere. After 1 minute, the cooling bath was removed and neat chlorodiphenylphosphine (0.25 mL, 0.81 mmol) was added dropwise. The mixture was further stirred at room temperature for 1 hour. A saturated NH_4_Cl solution (10 mL) was then added for quenching. The organic phase was separated, dried over anhydrous Na_2_SO_4_ and the solvent was removed under reduced pressure at 40 °C. The raw product was chromatographed (silica gel 0.040–0.063 mm, Merck) employing a mixture of CHCl_3_/MeOH = 99/1 as eluting solvent. Yield: 100 mg (0.16 mmol, 60% yield). ^1^H-NMR (CDCl_3_) δ: 2.51 (3H, s, N-CH_3_), 3.12 (3H, s, 2-OCH_3_), 3.50 (3H, s, 1-OCH_3_), 3.55 (3H, s, 9-OCH_3_), 3.85 (1H, d, *J =* 4.9 Hz, 2-OCH_2_O-), 3.88 (3H, s, 10-OCH_3_), 4.78 (1H, d, *J =* 4.9 Hz, 2-OCH_2_O-), 5.28 (2H, s, 9-OCH_2_O-), 7.06 (1H, s, H-8), 7.40–7.55 (6H, m, Ar-H_meta_ + Ar-H_para_), 7.67 (1H, dd, *J =* 7.8, 1.4 Hz, Ar-H_ortho_), 7.70 (1H, dd, *J =* 7.8, 1.5 Hz, Ar-H_ortho_), 7.78 (1H, dd, *J =* 7.5, 1.5 Hz, Ar-H_ortho_), 7.81 (1H, dd, *J =* 7.4, 1.6 Hz, Ar-H_ortho_), 7.97 (1H, s, H-11); ^13^C-NMR (CDCl_3_) δ: 27.8 (C-4), 33.6 (C-7), 43.6 (N-CH_3_), 52.6 (C-5), 56.2 (10-OCH_3_), 56.3 (9-OCH_3_), 57.2 (2-OCH_3_), 60.2 (1-OCH_3_), 63.3 (C-6a), 95.2 (9-OCH_2_O-), 99.1 (2-OCH_2_O-), 112.6 (C-11), 115.0 (C-8), 121.3 (C-3), 124.7 (C-11a), 128.2 (Ar-C_meta_), 128.3 (Ar-C_meta_), 128.4 (Ar-C_meta_), 128.5 (Ar-C_meta_), 130.1 (C-3a), 130.6 (Ar-C_ortho_), 130.7 (Ar-C_ortho_), 130.8 (Ar-C_para_), 131.4 (2C, Ar-C_para_ + Ar-C_ortho_), 131.5 (Ar-C_ortho_), 132.4 (C-1a), 133.9 (O=P-C), 135.0 (O=P-C), 146.6 (C-9), 147.5 (C-1), 148.2 (C-10), 151.9 (C-2); ^31^P-NMR (CDCl_3_) δ: 29.1; HRMS (CI) Found: m/z 616.2481 (M+H^+^), Calcd. for C_35_H_39_NO_7_P: m/z 616.2459.

### 3.3. Cell Lines

The experimental cell cultures were obtained from the American Type Culture Collection (Rockville, MD, USA). MCF-7 and MDA-MB-231 cells were grown in DMEM-F12 medium containing 10% FCS, 100 U/mL penicillin, 100 µg/mL streptomycin and 1 mM glutamine. DHF dermal human fibroblast cells were grown in DMEM-F12 medium containing 10% FCS, 100 U/mL penicillin, 100 µg/mL streptomycin and 1 mM glutamine. Cells were seeded into 96 well microtiter plates in 100 µL at plating density of 3 × 10^3^ cells/well. After 24 h of incubation at 37 °C in a humidified 5% CO_2_: 95% air mixture to allow cell attachment, the cells were treated with different concentrations of drugs [boldine (**1**) and derivatives **2–4**] and incubated for 72 h under the same conditions. Stock solutions of compounds were prepared in ethanol and the final concentration of this solvent was kept constant at 1%. Control cultures received 1% ethanol alone.

### 3.4. In vitro Growth Inhibition Assay

The sulforhodamine B assay was used according to the method of Skehan *et al*. [8] with some modifications [9]. Briefly, the cells were set up 3 × 10^3^ cells per well of a 96-well, flat-bottomed 200 μL microplate. Cells were incubated at 37 °C in a humidified 5% CO_2_ / 95% air mixture and treated with the compounds at different concentrations for 72 hours. At the end of the drug exposure, cells were fixed with 50% trichloroacetic acid (TCA) at 4 °C (TCA final concentration 10%). After washing with distilled water, cells were stained with 0.1% sulforhodamine B, dissolved in 1% acetic acid (50 µL/well) for 30 min, and subsequently washed with 1% acetic acid to remove unbound stain. Protein-bound stain was solubilised with 100 µL of 10 mM unbuffered Tris base, and the cell density was determined using a fluorescence plate reader (wavelength 540 nm). Values shown are the % viability vs. ctrl + SD, with three independent experiments in triplicate.

## 4. Conclusions

The derivatization of boldine with a phosphinyl group, introduces a distinct capability as a potential drug for breast cancer treatment which is not found in the boldine itself nor in 2,9-dimethoxy-methylboldine or 2,9-dimethoxymethyl-3-bromoboldine. This capability, due to the phosphinyl group in **4**, may be attributed to the enhanced lipophilicity as well as an increasing intercalating behavior. This *in vitro* inhibitory effect in the growth of two breast cancer lines cells opens a promising way to introduce structural modifications in this natural product which enhances its biological activity.
